# Composition and diversity of 16S rRNA based skin bacterial microbiome in healthy horses

**DOI:** 10.1007/s11259-024-10444-7

**Published:** 2024-06-20

**Authors:** Viola Strompfová, Lucia Štempelová

**Affiliations:** Centre of Biosciences of the Slovak Academy of Sciences, Institute of Animal Physiology, Šoltésovej 4-6, 040 01 Košice, Slovakia

**Keywords:** Bacterial microbiome, Skin, Horse, 16S rRNA sequencing

## Abstract

Characterization of microbiota structure on the skin of healthy horses is important for further development of modulation strategies to ensure optimal bacterial composition for physiological processes. This requirement is also supported by the relatively high incidence of dermatological diseases in horses and thus the need to manage them therapeutically. The taxonomic analysis of skin samples (*n* = 30) from five different body parts of clinically healthy Shetlands ponies females (neck, back, abdomen, pastern, muzzle) kept under homogeneous conditions (in open stalls with paddock, feed with dry hay, green grass ad libitum and granulated feed) was performed using amplification of V3—V4 region of the 16S rRNA gene. Results indicate that bacteria associated with healthy equine skin represent 18 phyla, 29 classes and 119 families. The most abundant phyla were Proteobacteria (30.8 ± 9.1%) followed by Actinobacteriota (20.4 ± 7.6%), Firmicutes (19.5 ± 10.1%), Bacteroidota (8.5 ± 5.0%) and Deinococcota (7.2 ± 14.8%). Among 229 genera identified, *Corynebacterium* (7.4 ± 6.5%) was the most abundant genus in skin sites of horses, followed by *Deinococcus* (7.1 ± 14.9%) and *Macrococcus* (5.0 ± 8.2%). Indices for the richness and diversity of species within bacterial populations for five regions of horses skin revealed no significant variations observed for species richness (Chao1, *p*-value 0.2001) but significant result for species evenness (Shannon, *p*-value 0.0049) with maximum on the neck and minimum on the back skin site. The clustering was seen across samples from different skin sites but also across samples collected from individual horses.

**Supplementary Information:**

The online version contains supplementary material available at 10.1007/s11259-024-10444-7.

## Introduction

The skin surface is a unique and variable ecosystem providing variable physiological conditions to microorganisms. In general, these skin conditions represent an inhospitable site for microorganisms because the skin is acidic, high-salt, colder and dry environment covered with antimicrobial peptides and constantly shedding superficial layers (Percival et al. 2012). The roles of microbes present in this unique habitat span from pathogenic, symbiotic to harmless forms (Costello et al. 2009; Ursell et al. 2012). While the gut microbiome has been investigated extensively (Schmitz and Suchodolski 2016; Sandri et al. 2017), the skin microbiome becoming another focus of research more recently (O'Shaughnessy-Hunter et al. 2021). Skin, the largest organ of the human body, is second only to the gut in terms of bacterial density, with an approximate density of 10^4^ to 10^6^ bacteria per square centimetre and over 200 genera characterised (Cundell 2018). According to some authors, diversity of the skin microbiota may be higher than diversity of gut microbiota (Grice and Segre 2011). Within the integumentary system, a growing interest in studying the taxonomic composition of the microbiota in different animals in the last decade is noted (Ross et al. 2019). Recent especially human studies have shown that the skin microbiota plays a fundamental role in in the induction, education, and function of the host immune system and in protection against skin pathogens (Byrd et al. 2018; Meisel et al. 2018). Skin bacteria interacts with host keratinocytes and innate immune system what stimulates production of effector molecules including cytokines, chemokines and antimicrobial peptides (Gallo and Nakatsuji 2011; Meisel et al. 2018). Thus, symbiotic microorganisms help to maintain the skin barrier and homeostasis, metabolize natural products, produce antimicrobial proteins, and influence the natural course of many skin diseases (Iwase et al. 2010; Belkaid and Segre 2014). It is still unknown how exactly homeostasis is maintained by the skin microbiota, but it is well known that the balance among members of the skin microbial communities plays a key role in protection against skin disorders (Zhu et al. 2023). Imbalances in the skin microbiota composition (dysbiosis) can significantly impact the immune system and are e.g. in humans associated with several skin conditions, either pathological such as acne, eczema, allergies or dandruff or non-pathological such as sensitive skin, irritated skin or dry skin (Sfriso et al. 2020). Therefore, it is important to know which bacterial taxa are „Normal “ and which are involved in pathological process to further investigate optimal therapeutic strategies to restore microbial balance. Although the most of taxonomic studies have been carried out in dogs and cats, rare study of O'Shaughnessy-Hunter et al. (2021) using sequencing method in horses indicated seasonal variation in their skin microbiota. They identified 38 phyla and 1,665 genera whereby the most common phyla across all samples were Proteobacteria, Firmicutes, Actinobacteria and Bacteroidetes. However, their abundance on various body sites were different. Since there is a lack of studies monitoring the taxonomic composition of the bacteriome on the healthy skin of horses using next-generation sequencing, we decided to find out abundance of bacterial taxa distributed on five body sites (neck, back, abdomen, pastern, muzzle) of healthy horses sharing the same environmental conditions and feeding the same diet. Namely, microbial community structure in different body sites can differ greatly and determine which genera will be involved in the infection in case of injury. According Wang et al. (2021), the relative abundances of potential pathogenic bacteria also differed in different body locations, providing a foundation for studying skin-associated bacterial diseases.

## Material and methods

### Samples collection

The superficial skin swabs (*n* = 30) were obtained from horses (Shetland ponies, 17.7 ± 2.2 years old) located in close area of the city Košice (Kavečany, Slovakia) in cooperation with breeding inspector of Zoo in May 2023. Horses are kept in open stalls with paddock. They were feed with dry hay, green grass (both ad libitum) and granulated feed Mazuri Zebra pellets (Mazuri, USA, 150 g per individual). It has the following composition: ground oats, wheat middlings, dehydrated alfalfa meal, oat hulls, linseed meal, dried beet pulp, dehulled soybean meal, salt, vitamin and mineral premix. Skin swabs were collected from five body areas during a routine clinical examination by a veterinarian. Sampling sites from equine skin included neck, dorsal back, abdomen, pastern and muzzle. All horses were clinically healthy and had no history of antimicrobial exposure within the preceding three months. Samples were obtained by rubbing each area using FLOQSwabs (Copan, Italy) soaked in sterile SCF-1 solution (50 mM Tris buffer, 1 mM EDTA and 0.5% Tween-20) and the collection tube was filled with 2 ml of the stablization solution DNA/RNA Shield R1100-250 (Zymo Research, Irvine, USA). To prevent cross-contamination, the person performing sampling wore a pair of sterile gloves for each individual. The samples were than frozen at -20 °C until processing. For each animal the questionnaire (name, breed, sex, age, locality, time spent outside) was completed.

### DNA isolation and sequencing

Samples were processed as a part of the Sequencing service in BioVendor Group (BioVendor, Czech Republic), including the collection tube preparation, DNA extraction, sequencing library preparation, and bioinformatic analysis. DNA was extracted using the CatchGene Stool DNA Kit (CatchGene, Taiwan), following the manufacturer's manual. The 16S rRNA library was prepared according to the Illumina 16S Metagenomic Sequencing Library Preparation Protocol with some deviations. The V3-V4 region of the 16S rRNA was amplified using the previously published degenerated primers (Klindworth et al. 2013) with the spacer sequences to increase the library diversity (Fadrosh et al. 2014). The indexing PCR was carried out with the adapters including the 8-bp long indexes. For both PCR, the Q5 High-Fidelity polymerase (New England BioLabs, USA) was used, and PCR products were purified using the Agencourt AMPure XP beads (Beckman Coulter Genomics, USA) according to the manufacturer’s recommendations. Qubit dsDNA HS Assay Kit (Invitrogen, USA) together with the Infinite® 200 PRO microplate reader (Tecan, Switzerland) and Tecan i-control™ software were used to assess the concentration of purified PCR products, and the gel electrophoresis (2% gel) was performed to control the amplicon lengths distribution. Several PCR products were analysed using the Fragment analyser Femto Pulse System (Agilent Technologies, USA) and Ultra Sensitivity NGS Kit (Agilent Technologies), to verify the length distribution and quality of products. Differently indexed products were equimolarly pooled according to the assessed concentrations and the library was diluted to a final concentration of 12 pM, and 15% of PhiX DNA (Illumina, USA) was added. Sequencing was performed with the MiSeq Reagent Kit v3 (600-cycle) a MiSeq sequencing instrument according to the manufacturer’s instructions (Illumina, USA). Pair-end reads passing quality control were demultiplexed, barcodes and primers were trimmed using cutadapt software version 3.4 (Martin 2011). ASVs (Amplicon Sequence Variants) were constructed using the DADA2 algorithm (Callahan et al. 2016), chimeras were excluded from further analysis. Taxonomy was assigned to each ASV based on the SILVA 123 reference database version 138.1

### Data analysis

MicrobiomeAnalyst platformwas used to perform the statistical analyses and visualisation of data (Chong et al. 2020). MicrobiomeAnalyst comprises four modules, and we used the Marker Data Profiling (MDP) module that is designed for analysis of 16S rRNA marker gene survey data. Before downstream analysis of alpha and beta diversity, the ASV table was filtered to remove spurious ASVs (20% of prevalence in samples). Alpha diversity analysis assesses the diversity within a sample. Two alpha diversity parameters Chao1 (species richness) and Shannon (species evenness) were used for comparison of samples. All comparisons were done through a T-test /ANOVA (Analysis of Variance) at the level of genera (phylogenetic groups). The beta diversity (comparison of the phylogenetic composition of the bacterial communities of the different samples) was studied based on relative abundance data analyzed by non-metric multidimensional scaling (NMDS) based on a Bray–Curtis similarity matrix. Statistical method ANOSIM was applied to evaluate the extent of variable effect on the dissimilarity of microbial communities. An R value near 1 means that there is dissimilarity between the groups, while an R value near 0 indicates no significant dissimilarity between the groups. Statistical method pairwise PERMANOVA was applied to compare results between two groups of samples. Throughtout the article, a *p*-value of 0.05 or lower was considered as statistically significant. Only in the section where abundances of bacterial phyla and genera were compared among individual parts of the body resulting *p-*values were additionally controlled for multiple comparisons using False Discovery Rate (FDR).

## Results

**Fig. 1:**
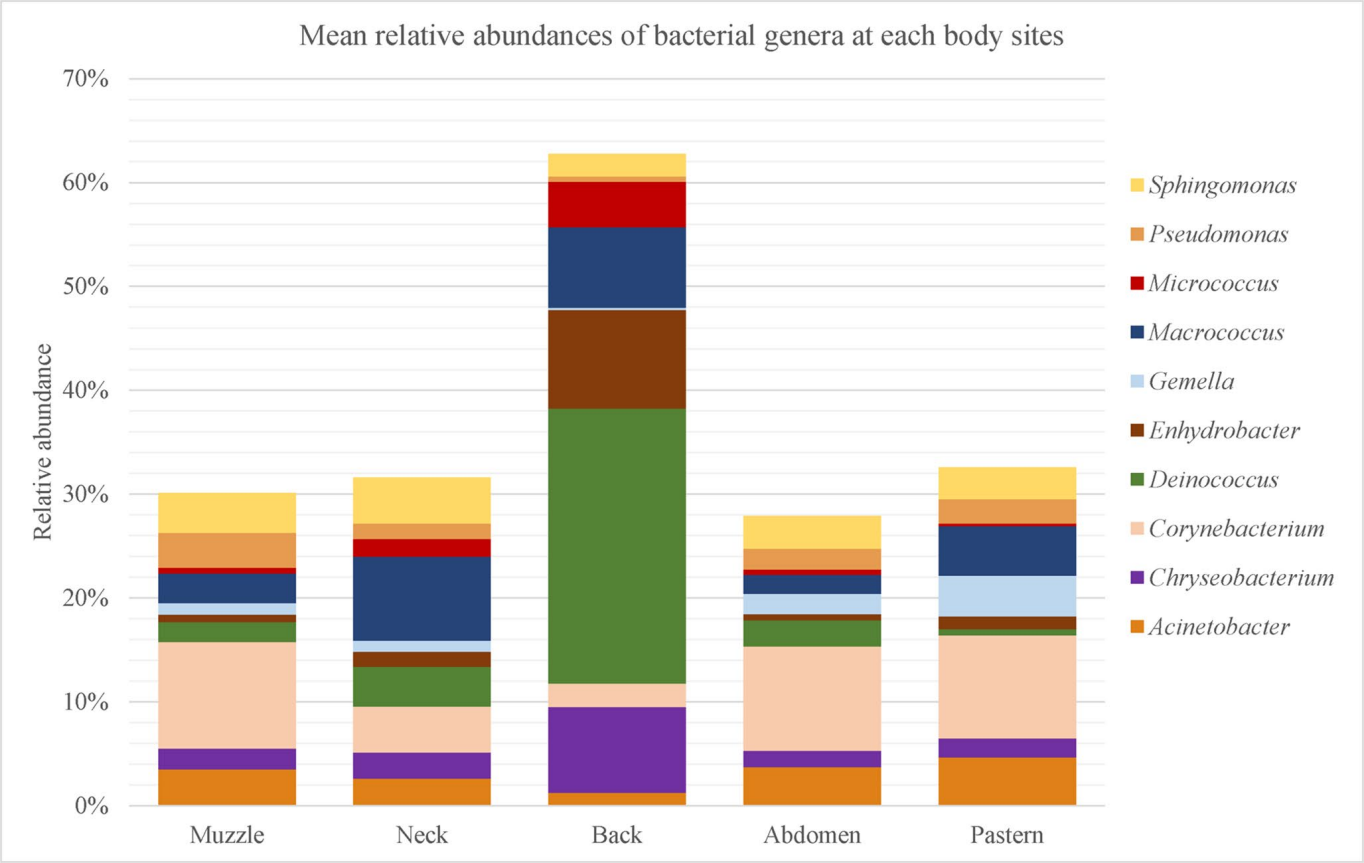
Taxonomic composition per skin site at genus level. Bar graphs show the mean relative abundances of top 10 bacterial genera at each body site (muzzle, neck, back, abdomen, pastern). Significant differences corrected using FDR were observed only in *Deinococcus* (pastern vs. back, FDR: 0.0461) *Pseudomonas* (abdomen vs. back, FDR: 0.0461; back vs. muzzle, FDR: 0.0113; pastern vs. back, FDR: 0.0439) and *Gemella* (pastern vs. back, FDR: 0.0461)

**Fig. 2:**
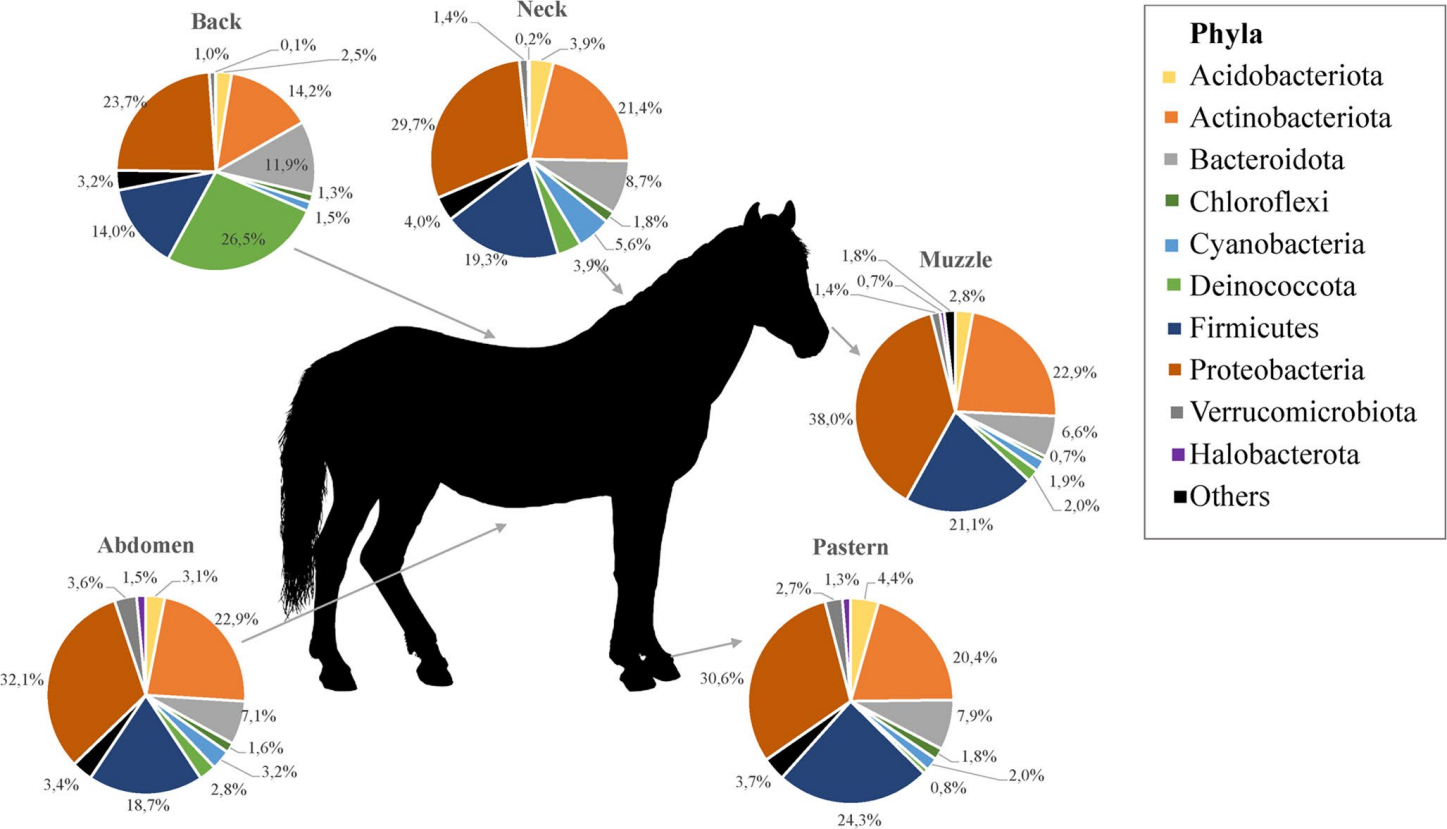
Taxonomic composition per skin site at phylum level. Pie charts show the mean relative abundances of top 10 bacterial phyla at each body site (muzzle, neck, back, abdomen, pastern)

The total number of sequence reads from samples was 3,855,338 with a median of 131,617 sequences per sample. Our results revealed a total 18 phyla, 29 classes and 119 families. The overall percentage of the five body sites combined revealed that the most abundant phyla were Proteobacteria (mean relative abundance: 30.8 ± 9.1%) followed by Actinobacteriota (20.4 ± 7.6%), Firmicutes (19.5 ± 10.1%), Bacteroidota (8.5 ± 5.0%) and Deinococcota (7.2 ± 14.8%). At the family level, Corynebacteriaceae (Phylum Actinobacteriota, Class Actinobacteria, order Corynebacteriales) were the most abundant, representing 7.4 ± 6.5% of the total taxa identified. Deinococcaceae (7.1 ± 14.9%), Staphylococcaceae (7.0 ± 8.3%) and Moraxellaceae (6.4 ± 5.5%) also were commonly found. Among 229 genera identified, *Corynebacterium* (7.4 ± 6.5%) was the most abundant genus in skin sites of horses, followed by *Deinococcus* (7.1 ± 14.9%) and *Macrococcus* (5.0 ± 8.2%, Fig. 1). As concerned skin site, Proteobacteria were predominant in all skin sites (23.7 ± 10.4% – 38.0 ± 7.3%) except back where Deinococcota phylum (26.5 ± 24.5%) was the most abundant. Actinobacteriota was occured in all sites, ranged from 14.2 ± 7.1% (back) to 22.9 ± 4.5% (muzzle). Firmicutes was the most abundant in pastern and muzzle region (24.3 ± 10.1% and 21.1 ± 7.7%, respectively) whereas it was on the back in lower abundace (14.0 ± 14.6%, Fig. 2).

Analysis of distribution of bacterial genera on skin sites revealed that *Macrococcus* was predominant on neck (8.1 ± 7.1%). *Deinococcus* was the most abundant genus on back (26.5% ± 24.5%) whereas *Corynebacterium* was predominant on pastern (9.9 ± 6.4%), muzzle (10.3 ± 5.8%) and abdomen (10.0 ± 8.1%). Statistical analysis was done to compare abundances of bacterial phyla and genera among individual skin sites. When the phyla with the highest mean relative abundance were considered (top five), significant variations corrected using FDR were observed in Deinococcota (abdomen vs. back, FDR: 0.0007; back vs. neck, FDR: 0.0129; back vs. muzzle, FDR: 0.0002; pastern vs. back, FDR < 0.0001) and Bacteroidota (abdomen vs. back, FDR: 0.0292; back vs. muzzle, FDR: 0.0165; pastern vs. back, FDR: 0.0202). Among the most abundant ten genera, significant variations corrected using FDR were observed only in *Deinococcus* (pastern vs. back, FDR: 0.0461) *Pseudomonas* (abdomen vs. back, FDR: 0.0461; back vs. muzzle, FDR: 0.0113; pastern vs. back, FDR: 0.0439) and *Gemella* (pastern vs. back, FDR: 0.0461).

**Fig. 3:**
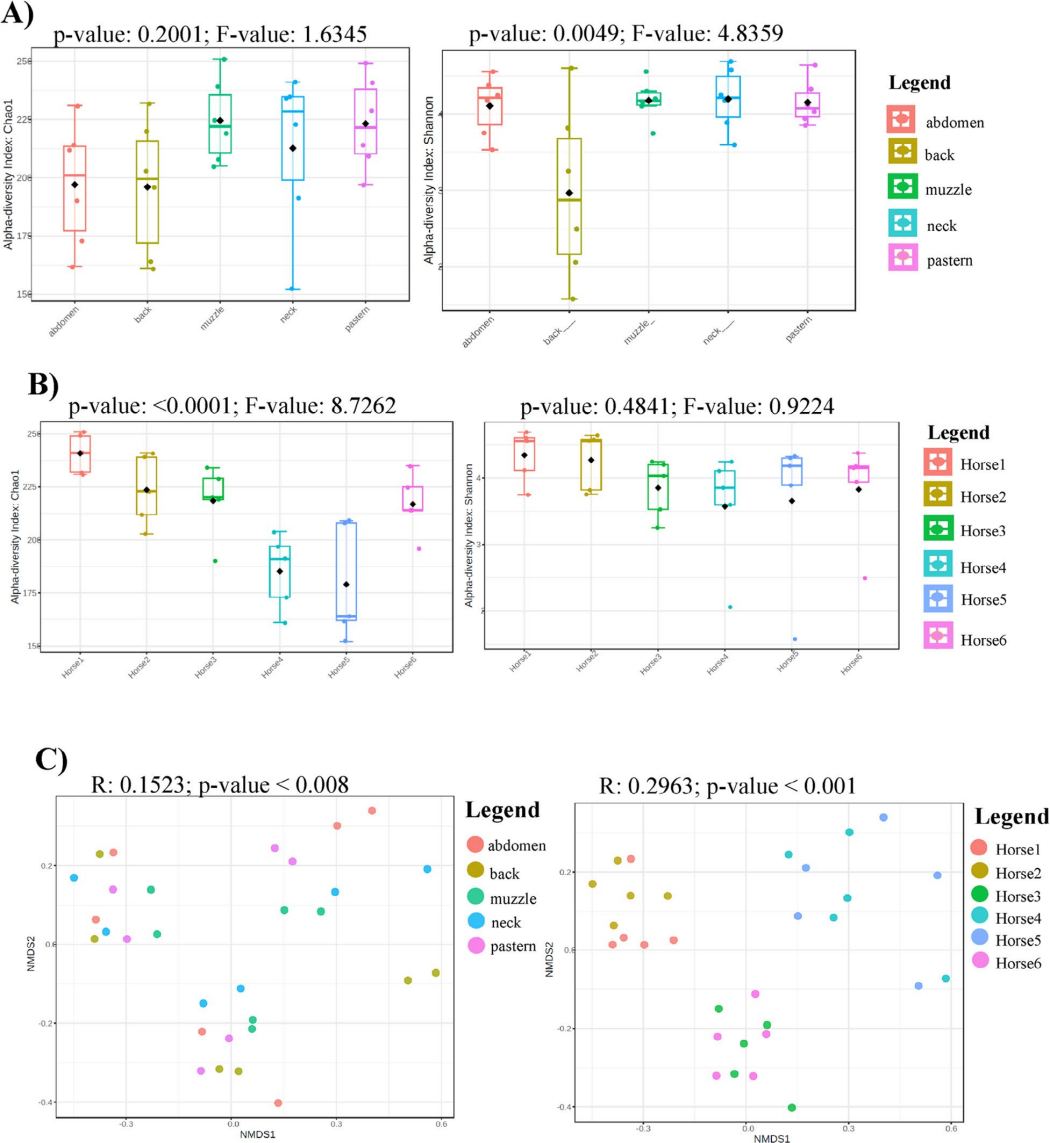
**A** Box-plots of alpha diversity indices Chao1 (left) and Shannon (right) for the five studied body sites of healthy horses. A *p*-value of 0.05 or lower was considered as statistically significant. No significant differences were obtained for species richness (Chao1, *p*-value 0.2001). Significant differences were detected for species evenness (Shannon, *p*-value 0.0049) with maximum on the neck and minimum on the back skin site (*p*-value 0.0453). Significat differences were seen also between back and the muzzle (*p*-value 0.0472). A higher F-value in Shannon index indicates that the variance in alpha diversity among groups is significantly greater than the variance within groups. **B** Box-plots of alpha diversity indices Chao1 (left) and Shannon (right) of the skin microbiota of six healthy horses. A *p*-value of 0.05 or lower was considered as statistically significant. No significant differences were detected for Shannon index. Significant differences were obtained for species richness (Chao1) among individial horses (Horse2 vs Horse4 *p*-value:0.0083; Horse2 vs Horse5 *p*-value:0.0181; Horse3 vs Horse4 *p*-value:0.0179; Horse3 vs Horse5 *p*-value:0.0303; Horse3 vs Horse1 *p*-value:0.0408; Horse4 vs Horse6 *p*-value:0.0171; Horse4 vs Horse1 *p*-value: < 0.0001; Horse5 vs Horse6 *p*-value:0.0336; Horse5 vs Horse1 *p*-value:0.00516; Horse6 vs Horse1 *p*-value:0.0177). **C** Beta diversity: Community structure profiling of bacterial communities across samples collected from 5 skin sites (left) and across skin samples from individual horses (right) are shown on 2D NMDS plots. Significant differences among groups are observed in both cases. The higher R-value on right plot indicates greater differences in genus composition among the studied groups

Core microbiome for all samples was composed of Proteobacteria, Actinobacteria, Firmicutes and Bacteroidota (prevalence 1.0 for all mentioned phyla). Micrococcaceae (1.0), Sphingomonadaceae (0.97), Moraxellaceae (0.97), Staphylococcaceae (0.93), Planococcaceae (0.93) were the core families with highest prevalence. *Sphingomonas* (0.93), *Corynebacterium* (0.9) and *Chryseobacterium* (0.87) were among most prevalent core genera. Indices for the richness and diversity of species within bacterial populations for five regions of horses skin revealed no significant variations observed for species richness (Chao1, *p*-value 0.2001) but significant result for species evenness (Shannon, *p*-value 0.0049, Fig. 3a) with maximum on the neck and minimum on the back skin site (*p*-value 0.0453). Significant differences were also observed between the back and the muzzle (*p*-value 0.0472). After comparison the bacterial composition among individual horses, we find significant variances only for Chao 1 (*p*-value: < 0.0001; Shannon: *p*-value: 0.4841, Fig. 3b).

We also performed beta diversity analysis which assesses the similarities among samples of the same community. While choosing skin location as an experimental factor, the results on NMDS plot revealed five partially overlapping clusters (R: 0.1523; *p*-value < 0.008, Fig. 3c). The used pairwise PERMANOVA showed significant differences between these groups of samples: muzzle vs. back (*p*-value: 0.007), back vs. abdomen (*p*-value: 0.009) and back vs. pastern (*p*-value: 0.013). In addition, significant differences were seen after choosing individual horses as an experimental factor (R: 0.2965; *p*-value < 0.001, Fig. 3c). Detailed information of the pairwise PERMANOVA results are summarized in the supplementary material 2.

## Discussion

The composition of skin microbiota as was reported for humans is determined by the genetics, environmental factors and the local microenvironment (Oh et al. 2016). It is believed that microbial taxonomic composition varies from one body part to another, therefore we decided to collect samples from five parts with different conditions for growth of bacteria. Grice and Segre (2011) demonstrated that colonization of bacteria is dependent on the physiology of the skin site, with specific bacteria being associated with moist, dry and sebaceous microenvironments. Specific bacteria are associated with sebaceous microenvironments such as lipophilic genus *Propionobacterium* sp. On the other hand, *Staphylococcus* and *Corynebacterium* spp. are the most abundant organisms colonizing moist areas, because these organisms prefer areas of high humidity. The most diverse skin sites in humans are the dry areas (Costello et al. 2009).

Concerning physiology of equine skin, there are differences in thickness of skin which decreases from dorsal to ventral with the forehead, dorsal neck, dorsal thorax, lumbosacral area and base of tail having the thickest skin (Talukdar et al. 1972). Hair coat thickness follows a similar pattern, decreasing dorsally to ventrally. As a result, skin temperature is highest on the head and trunk, decreasing distally (Scott and Miller 2011). The primary method of thermoregulation is, similar to humans, sweating. Whereas sweat glands of eccrine type are dominant in humans, sweat glands of horses are of the apocrine type producing viscous, milky secretion (Jenkinson et al. 2006). Equine sweat is extremely basic with pH 8.0–8.9 and thus has antimicrobial effects. We performed also measurement of skin pH in the sampled regions and it was between 7.07 ± 0.25 (pastern, neck) and 7.24 ± 0.26 (abdomen, unpublished data). Since many studies found association between cutaneous dysbiosis and inflammatory skin diseases it is important to first define what is considered normal microbiota of healthy skin. As it was previously mentioned, there is a lack of studies dealing with the microbiota of the skin of healthy horses and therefore the comparison of our results with other studies is difficult.

In our study, Gram-negative bacteria easily prevailed on equine skin in overall (Proteobacteria, Bacteroidota, Deinococcota) and the first most abundant phylum on majority of body parts was Gram-negative phylum Proteobacteria. Actinobacteria, Firmicutes, Deinococcota and Bacteroidota belonged to less abundant phyla and their relative abundance was different according to body site. In contrast, the human skin microbiota is quantitatively dominated by Gram-positive bacteria, detected by both culture and metagenomics (Cosseau et al. 2016). At the phylum level, Firmicutes and Actinobacteria are recognized as the major component of the skin microbiota in humans (Grice et al. 2008). As concerned Gram-negative taxa, they were generally considered to be of environmental origin (Cosseau et al. 2016). It seems environmental factors play an important role in microbial composition of the skin in many animal species. The reason why nonhuman mammals harbour especially environment-associated taxa is different lifestyle and more rare bathing compared to humans. When comparing to the only study of microbiota in healthy horses (O’Shaughnessy-Hunter et al. 2021), the most common phyla identified in horses are identical with our results. Similarly, they idenfied *Corynebacterium* and *Macrococcus* across all sites, but also further genera such as *Pseudomonas*, *Psychrobacter*, *Acinetobacter*, *Desemzia* or *Carnobacterium*. The exception is the phylum Deinococcota detected in our study, known as extremophiles, that are highly resistant to environmental hazards.

*Corynebacterium*, the most abundant genus in our study, is Gram-positive bacterium included in the phylum Actinobacteria (Bernard and Funke 2015). It consists a diverse group of bacteria including animal and plant pathogens, as well as saprophytes. They occur in nature (e.g. soils and water) and are often part of the normal human and animal skin flora and mucous membranes as commensals (Bernard and Funke 2015). Our results confirm their predominance on moist areas—muzzle and pastern what is consistent with its humidity requirements for their growth (Byrd et al. 2018). However, under certain circumstances, they can cause skin problems. In horse, it was described case of *Corynebacterium* folliculitis manifesting by mildly pruritic multiple skin lesions that had progressed from nodules to alopecia and crusts (Heffner et al. 1988).

Interestingly, *Deinococcus* was the second most abundant genus on average across all samples with its predominance on the back skin site. The genus includes several species that are resistant to radiation, can decompose nuclear waste and other toxic materials and survive extremes of heat and cold (Battista et al. 1999). Tian et al. (2022) identified these bacteria in oral cavity of lizard *Japalura *sensu lato, what confirms its ability to live in extreme environments. The higher abundances of *Deinococcus* on the back in our study could be related to their higher resistance since this part of the body is most exposed to UV radiation and mechanical damage (saddle region). On the other hand, detection of gut microbiota profile showed increase of abundance of this bacteria in a chronic inflammatory disease such as ankylosing spondylitis in patients (Liu et al. 2022).

The third abundant genus was *Macrococcus*, a genus of Gram-positive cocci that are coagulase negative and catalase positive and belong to the family Staphylococcaceae. The study of Carroll et al. (2023) providing insight into evolution, population structure and functional potential of this genus reported their animal commensal, veterinary clinical, food-associated (including food spoilage), and environmental origins. Only 4.5% of sequenced genomes originated from human clinical cases. Since some of detected bacteria including *Macrococcus* carry antimicrobial resistance genes with possibility to transfer them to other microorganisms including taxa with a higher virulence potential, it is necessary to kept in mind this fact when we are in contact with these animals.

We observed significant differences in abundances of three bacterial genera among skin sites– *Deinococcus*, *Pseudomonas* and *Gemella. Pseudomonas* was detected in higher abundances on muzzle (3.3 ± 1.3%) and pastern (2.4 ± 1.4%). The higher abundance of *Pseudomonas* on the pastern in healthy horses could make this site as a predisposing area for the disease– equine pastern vasculitis. This dermatological disease is associated with multidrug-resistant *Pseudomonas aeruginosa* isolates (Panzuti et al. 2020).

Studies have also looked at how diverse skin microbiota is. The study of Cuscó et al. (2017) demonstrated that mucocutaneous perianal region and nasal skin of healthy dogs have lower alpha diversity values when compared to all other haired skin regions. Surprisingly, these results are not consistent with our current observation whereas we observed significantly lower alpha diversity values at back skin site compared to neck and muzzle. Similarly, study of O’Shaughnessy-Hunter et al. (2021) also showed lower alpha diversity values at back skin site in healthy horses, however these findings were not significant. The reasons for the lower bacterial diversity on back of horses may be that it is a place rich in sweat containing alkaline minerals, less exposed to the environment and often covered by the saddle. On the other hand, the muzzle area could be more diverse due to frequent direct contact with the horse's environment and food. Moreover, we observed significant differences for species richness (Chao1) among individial horses. It is an interesting finding wheares the horses included in the study came from the same environment and the influence of nutrition and external environment was minimal.

Results of beta-diversity showed important fact that the main force driving the skin microbiota composition is the individual (despite sharing of the same environment), followed by the skin site. Similarly, these two factors also shaped skin microbiota in humans with great variability within several skin sites of an individual and between individuals have been reported (Ursell et al. 2012; Oh et al. 2014). The study of Cuscó et al. (2017) characterizing skin microbiota in healthy homogenous Golden-Labrador Retriever dogs revealed also individual and skin sites differences. It seems, specific physicochemical conditions of microenvironment of anatomical locations in each host play important role in shaping the skin microbiota composition.

In conclusion, the present results are among first studies dealing with taxonomic composition of skin microbiota in healthy horses—Shetland ponies females identified using sequencing method. Our study revealed that the skin microbiota of healthy horses is diverse comprising also high abundance of Gram-negative Proteobacteria. Alpha analysis showed that bacterial diversity differs in individual skin sites. The lowest diversity was observed in the back area, the highest in the neck and muzzle area. Moreover, beta analysis of samples revealed a large inter-individual variability and differences among skin sites even in homogeneous group of animals. Further studies involving horses from different geographical regions bring more comprehensive data in this field.

### Supplementary Information

Below is the link to the electronic supplementary material.

Supplementary file1 (XLSX 107 kb)

Supplementary file2 (XLSX 17 kb)

## Data Availability

No datasets were generated or analysed during the current study.

## Declarations

### Accession number

Illumina MiSeq sequencing data are avaliable in BioProject SRA database uder accesion number PRJNA1113871.
